# In vitro alternative for reactogenicity assessment of outer membrane vesicle based vaccines

**DOI:** 10.1038/s41598-023-39908-7

**Published:** 2023-08-04

**Authors:** Marijke W. A. Molenaar-de Backer, Paulien Doodeman, Fereshte Rezai, Lisa M. Verhagen, Arno van der Ark, Els M. Plagmeijer, Bernard Metz, Naomi van Vlies, Olga Ophorst, René H. M. Raeven

**Affiliations:** 1 Department of Virology and MAT Services, Sanquin Diagnostiek, Plesmanlaan 125, 1066CX Amsterdam, The Netherlands; 2 Intravacc, Antonie Van Leeuwenhoeklaan 9, 3721 MA Bilthoven, The Netherlands

**Keywords:** Vaccines, Drug safety

## Abstract

Intrinsic or added immune activating molecules are key for most vaccines to provide desired immunity profiles but may increase systemic reactogenicity. Regulatory agencies require rabbit pyrogen testing (RPT) for demonstration of vaccine reactogenicity. Recently, the monocyte activation test (MAT) gained popularity as in vitro alternative, yet this assay was primarily designed to test pyrogen-free products. The aim was to adjust the MAT to enable testing of pyrogen containing vaccines in an early stage of development where no reference batch is yet available. The MAT and RPT were compared for assessing unknown safety profiles of pertussis outer membrane vesicle (OMV) vaccine candidates to those of Bexsero as surrogate reference vaccine. Pertussis OMVs with wild-type LPS predominantly activated TLR2 and TLR4 and were more reactogenic than Bexsero. However, this reactogenicity profile for pertussis OMVs could be equalized or drastically reduced compared to Bexsero or a whole-cell pertussis vaccine, respectively by dose changing, modifying the LPS, intranasal administration, or a combination of these. Importantly, except for LPS modified products, reactogenicity profiles obtained with the RPT and MAT were comparable. Overall, we demonstrated that this pertussis OMV vaccine candidate has an acceptable safety profile. Furthermore, the MAT proved its applicability to assess reactogenicity levels of pyrogen containing vaccines at multiple stages of vaccine development and could eventually replace rabbit pyrogen testing.

## Introduction

Systemic reactogenicity following immunization is a commonly observed and monitored adverse effect. The reactogenicity is caused by pathogen-associated molecular patterns (PAMPs) that are present in the vaccine. Whereas some vaccines are designed to be completely free of PAMPs to minimize systemic reactogenicity, other vaccine designs use the adjuvating effect of intrinsic or added PAMPs to steer immune responses towards a specific immune profile^1^. Outer membrane vesicles (OMVs) are small particles that are shedded from the outer membrane of Gram-negative bacteria and therefore contain intrinsic PAMPs such as lipopolysaccharide (LPS) and lipoproteins^2^. Over the last decades, OMVs have been implemented in vaccines developed against different bacterial species, such as *Bordetella pertussis* (*B. pertussis*), *Neisseria meningitidis* serogroup B (*NmB*)*, Neisseria gonorrhoeae* (*Ng*) and *Shigella*^3–7^. Moreover, OMVs can be applied as carrier and adjuvant for protein vaccines against i.e., viral infections, such as SARS-CoV-2^8^. The composition of PAMPs in OMVs, and therefore the reactogenicity profile, can differ as a result of selected species, production process or genetic modifications^9^. The ability of assessing systemic reactogenicity in an early stage of vaccine development is therefore key to design pyrogen containing vaccines with a balanced profile of high immunogenicity and acceptable reactogenicity.

All parenteral products, including OMV vaccines must be tested for pyrogen/endotoxin content, to ensure an acceptable safety profile. Currently there are several methods for pyrogen/endotoxin testing. The bacterial endotoxin test (BET) can only test for endotoxins and would therefore fail to detect other pyrogens present in OMVs, e.g., lipoproteins. The other method is the rabbit pyrogen test (RPT) which can detect endotoxin and non-endotoxin pyrogens. However, the RPT is modified to analyse inherent pyrogenic vaccines (e.g., the product is diluted before intravenous injection, or instead, is injected intramuscularly)^10^. In addition, recent studies showed that the RPT can be less sensitive to endotoxin than assumed, which probably depends on the type of rabbit breed and strain used^11^. The most recent method to detect pyrogens is the monocyte activation test (MAT) which can detect all pyrogens and not only endotoxins, as is the case in the BET. In addition, the MAT better reflects the human immune system since it uses human monocytes instead of those from the rabbit immune system. For parenteral medicines, the MAT is a compendial method in the European Pharmacopoeia (Ph. Eur.) and is seen as the preferred replacement method for the RPT by the EU commission to comply with the 3R principle of replacement, reduction and refinement and sustainability goals^12^.

For vaccines that contain intrinsic or added PAMPs, a delicate balance between safety and immunogenicity is required; too much reactogenicity will lead to more (severe) side effects and too little reactogenicity or stimulation will lead to reduced or no efficacy (immunity). The MAT can be used to assess this level of reactogenicity and is currently used as a safety and consistency test for Bexsero at release^13^. For testing OMV based vaccines that are in the development phase, the MAT required optimization to be able to assess the reactogenicity of these OMVs with a single general MAT procedure. This study describes the results of the optimization process and MAT testing of several OMVs from different bacterial species. The MAT format used was a reference lot comparison, which is in line with current Ph. Eur. chapter 2.6.30 (07/2017). For a reference lot comparison, a known and justified reference lot should be used. However, since our OMVs are still in the development phase it is not straightforward to indicate a reference lot. Therefore, Bexsero was chosen as a surrogate reference vaccine since this OMV-based vaccine has an accepted safety profile and MAT data obtained with Bexsero is available in the literature^13–15^. Finally, the unknown safety profiles of a selection of candidate pertussis OMV vaccines were unravelled and compared head-to-head in the MAT and the RPT demonstrating that the MAT can also be applied in this early phase of vaccine development.

## Materials and methods

### OMV products

All OMVs were produced using a downscaled platform production process developed at Intravacc^16^ with an EDTA extraction method and some optimization steps depending on type of OMV and species. Two types of OMVs against *B. pertussis* were included either derived from the B1917 strain (containing unmodified LPS) (named omvPV(WT LPS) hereafter) or a strain with a LpxA modification in the LPS^17^ (named omvPV(LpxA) hereafter). Details on the three different production processes that were used to produce omvPV(LPS) with a higher yield and purity could not be disclosed. The meningococcal OMV vaccine was isolated from a nonencapsulated *Neisseria (N.) meningitidis* serogroup B H44/76 strain with a Lpxl1 modification^18^ (named omvNmB(Lpxl1) hereafter). The *Neisseria gonorrhoeae* strain MS11 with an added Lpxl1 modification was used to produce the gonococcal OMV vaccine (named omvNg(Lpxl1) hereafter). Total protein concentration was determined using a BCA method (Micro BCA™ Protein Assay Kit, Thermo Fisher) was used as standard vaccine dosing for all OMVs unless stated otherwise. The standard quality control of OMVs based on particle size, protein composition and LPS content was performed as described previously^19^, but data was not included. OMV structure was previously investigated using TEM^20^.

### LPS isolation and quantification

LPS was isolated from heat-inactivated *B. pertussis* lysate using a hot phenol-water extraction method^21^. LPS concentration was determined by analysing fatty acid composition with a gas chromatography method^22^. A molecular weight of 4057 g/mol or 4001 g/mol was used to calculate the LPS-concentrations for WT LPS or LpxA, respectively.

### Reference vaccines

Bexsero (GSK) and aPV (Daptacel DTaP, Sanofi Pasteur) were obtained from the international pharmacy. The whole-cell pertussis vaccine (wPV) (Pertussis Vaccine (Whole Cell) WHO International Standard (94/532)^23^ was obtained from The National Institute for Biological Standards and Control (NIBSC, England).

### MAT execution: cell culture

The MAT was performed using the MAT Cell Set (Sanquin Reagents, Amsterdam, The Netherlands), following manufacturer’s instructions with adaptations as described. In short, OMV preparations were diluted in complete medium (Iscove's Modified Dulbecco's Medium (IMDM; Lonza, Verviers, Belgium) supplemented with either 5% fetal bovine serum (FBS) or 5% human serum (HS) (both from Biowest, Nuaillé, France) as serum source) and 100 μL was added to wells of 96-wells plate. Each sample dilution was tested in duplo, triplicate or quadruplicate as indicated in each figure. Subsequently, a vial containing 5 × 10^6^ (± 1 × 10^6^) cryopreserved PBMCs (1 mL) was thawed in a water bath of 37 °C until a little clump of ice remained. The cell suspension was subsequently transferred to a new tube and diluted with 10 mL complete medium. In the MAT cell culture plate, 100 μL of cells was added to 100 μL sample. Subsequently, the 96-well cell culture plate was incubated in a humidified incubator at 37 °C in the presence of 5% CO_2_ for a duration of 18–24 h, with approximately 45.000 cells/well. In this study different batches of cryopreserved PBMCs (each with 4 different donors), HS, and OMV preparations were used.

### MAT execution: IL-6 ELISA

After overnight incubation, the cell culture supernatants were harvested and analysed in 1:5 dilution for the presence of IL-6 using a commercially available enzyme-linked immunosorbent assay (ELISA) kit (PeliKine Compact human IL-6 ELISA kit, Sanquin, Amsterdam, The Netherlands) combined with the additional ELISA buffers and reagents (PeliKine Toolset 1, Sanquin, Amsterdam, The Netherlands) following manufacturer’s instructions. Depicted are the optical density (OD) at 450 nm with subtraction of the background OD at 550 nm.

### Report cell lines

All cell lines used were obtained from Invivogen (France). HEK-Blue hTLR2, HEK-Blue hTLR3, HEK-Blue hTLR4, HEK-Blue hTLR7, HEK-Blue hTLR9, HEK-Blue hNOD1, HEK-Blue hNOD2 as well as control cell lines HEK-Blue Null1 and HEK-Blue Null2 were cultured (37 °C, 5% CO_2_) in Dulbecco’s Modified Eagles Medium (DMEM) (Gibco) supplemented with 100 µg/ml Normicin (Invivogen), 100 units penicillin, 100 units streptomycin, 2.92 mg/mL L-glutamine (Gibco), 10% heat-inactivated FBS (Gibco) and cell line-specific selective antibiotics (Invivogen) in a T75 flask. When 70–80% confluency was achieved, HEK-Blue cells were detached from culture flasks using PBS (Gibco) and sub-cultured. When passage 20 was achieved, cells were discarded.

HEK-Blue cells were plated in 96-well plates (flat-bottom) at 50.000 viable cells in 100 µL medium per well and cultured overnight (37 °C, 5% CO_2_). Cells were stimulated the next day with 100 µL OMVs starting at 2 µg/mL in fivefold serial dilutions and TLR/NOD antagonists as positive controls. Subsequently, plates were incubated for 24 h (37 °C, 5% CO_2_). Next, 20 µL culture supernatant of each well was transferred to wells in a 96-well plate containing 180 µL of pre-warmed Quanti-Blue detection reagent. Absorbance was read after 1 h at 630 nm with a microplate reader (Bio-Tek). TLR activation was determined by calculating the fold change over the unstimulated cells.

### Rabbit pyrogen test

50 healthy female rabbits (Outbred/New Zealand White) of 10–13 weeks old were included for the rabbit pyrogen test performed at a CRO (Charles River Laboratories Ireland Ltd., Ireland). The study plan was reviewed and approved by the Ethics Committee of Charles River Laboratories Ireland Ltd. The animal experiments were performed in compliance with the European Directive for the Protection of Animals used for Scientific Purposes, Directive 2010/63/EU, as transposed into Irish law under Statutory Instrument S.I. No 543 of 2012 and ARRIVE guidelines. After 3 days acclimatization, each animal had a telemetry temperature chip surgically implanted under anaesthesia (injectable anesthesia in the form of Medetomidine (Medetor 1 mg/ml) at 0.25 mg/kg, Ketamine (Narketan 100 mg/ml) at 15 mg/kg and Meloxicam (Metacam 2 mg/ml) at 1.0 mg/kg) into the abdominal cavity (intraperitoneal). Baseline temperature for each rabbit was determined over a period of 5 consecutive days (Study Day − 5 to Study Day 0) and had to be within normal limits (38–40 °C). Rabbits were randomly distributed over 10 groups (n = 5 per group) and housed together in a cage with 5 rabbits per pen. On Study Day 0, 7 groups (35 animals) were immunized 0.5 ml (0.25 ml at two separate sites) of the test item by the intramuscular route. Group 1: 50 µg omvPV(WT LPS), Group 2: 10 µg omvPV(WT LPS), Group 3: 50 µg omvPV(LpxA), Group 4: 10 µg omvPV(LpxA), Group 5: 1 dose Bexsero, Group 6: 1 dose wPV or Group 7: placebo. In addition, 3 groups (15 animals) were immunized by the intranasal route under anaesthesia (as described above) with 0.1 ml (0.05 ml per nostril) of the test item. Group 8: 50 µg omvPV(WT LPS), Group 9: 50 µg omvPV(LpxA) or Group 10: placebo. Subsequently the temperature of all 50 rabbits was monitored over a period of 30 h. In accordance with ARRIVE, animals were excluded from the study if they displayed unacceptable adverse effects post-surgery or as a result of immunization, e.g. visible signs of distress, refusing food and/or water or infection. A veterinarian would assess if these animals could be treated or if necessary, humanely euthanized. Overall, no animals or data points had to be excluded.

### Anti-pertussis specific IgG determination

The anti-pertussis IgG antibody levels in different HS batches were determined using a multiplex immunoassay as described previously^24^. In total, IgG antibody levels were determined against six purified pertussis antigens namely Bordetella resistance to killing (BrkA), Fimbriae 2/3 (Fim2/3), Filamentous hemagglutinin (FHA), Pertactin (Prn), Pertussis Toxin (Ptx), virulence associated gene 8 (Vag8). In addition, outer membrane vesicles derived from the B1917 strain were included to determine a broader range of antigens as these vesicles contain multiple pertussis antigens.

### Statistical analysis

GraphPad Prism 9.1.1 was used to plot the ODs against the dilution for graphical representation of the data; to determine the area under the curve (AUC) and to calculate the p-values. The AUC are depicted as mean ± SD. The p-values for MAT were calculated with Brown-Forsythe and Welch ANOVA test for multiple comparison, which assumes different SDs between groups; since SDs of omvPV(WT LPS and LpxA) and Bexsero were different. The RPT data was analysed with standard ANOVA for multiple comparison.

## Results

For the optimization of the MAT an OMV vaccine against *B. pertussis* (omvPV(WT LPS)) was used that was still in early development with respect to dosing, production process and modification to the LPS. An estimated dosing of 100 µg/mL total protein content in the OMV was used as starting dose based on immunogenicity data in mice^25^. Bexsero (OMV based vaccine against *Neisseria meningitidis* serogroup B (NmB)) was used as a reference product since the omvPV(WT LPS) vaccine was still in development and thus a reference lot was not yet available. The PBMCs used in this study were from a commercial kit (MAT Cell Set) and this kit contains cryopreserved pooled PBMCs from 4 different donors and the PBMCs were pre-screened to ensure reactivity to 5 different pyrogens, specifically HKSA (TLR 2), FLA-ST (TLR5), PAM3CSK4 (TLR2/1), PGN (Nod2) and endotoxin (TLR4).

### Comparison of the use of FBS or HS as serum source

**Figure 1:**
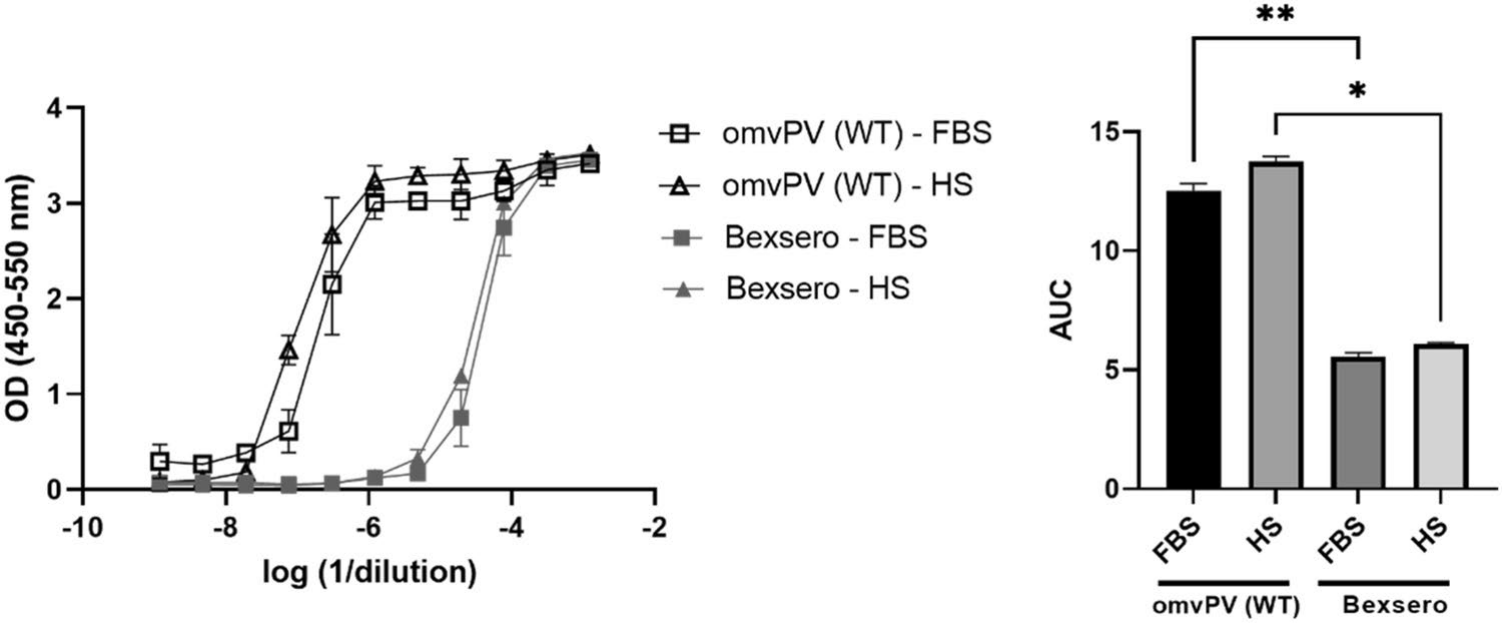
Comparison of HS and FBS in MAT. The IL-6 responses were compared when PBMCs were stimulated with different dilutions (concentrations) of omvPV(WT LPS) and Bexsero using either HS or FBS as serum source and is expressed as optical density (OD, left graph) and as area under the curve (AUC) of the OD curves (right graph). Undiluted omvPV(WT LPS) contained 100 μg/mL OMV and Bexsero contained 50 μg/mL OMV. After an overnight incubation, the IL-6 ELISA was performed on 1:5 diluted culture supernatants. Each sample was tested in duplicate. The AUC of the fitted curves is depicted as mean ± standard deviation (SD). *p < 0.05, **p < 0.01.

The PBMC based MAT requires addition of serum to the medium. Both fetal bovine serum (FBS) or human serum (HS) can be used for this and both were therefore compared when evaluating the reactogenicity of omvPV(WT LPS) and Bexsero. Eleven fourfold dilutions of omvPV(WT LPS) and Bexsero were tested to assess the most optimal range and serum source for the MAT. The tested sample dilutions ranged from 800x – 838,860,800x (corresponding to 1,600x – 1,677,721,600 × dilution in the well). For omvPV(WT LPS) there were no significant differences observed between the area under the curve (AUC) values of FBS-based and HS-based MAT (*p* value = 0.125) (Fig. 1). Moreover, the observed reactogenicity of Bexsero was also comparable between FBS-based and HS-based MAT (*p* value 0.306). The reactogenicity of omvPV(WT LPS) and Bexsero was significantly different (*p* < 0.05) in both FBS-based and HS-based MAT. Bexsero is less reactogenic than the tested dose of 100 µg/mL omvPV(WT LPS) (Fig. 1) as the IL-6 responses of Bexsero start to decrease from dilution 3,200 × onwards while for omvPV(WT LPS) this occurs from dilution 819,200 × onwards. Overall, no significant differences were observed between the FBS-based and HS-based MAT. Therefore, it was decided to continue with HS-based MAT as this better reflects the human immune system and is in line with the 3R principle.

### Robustness of HS-based MAT

**Figure 2:**
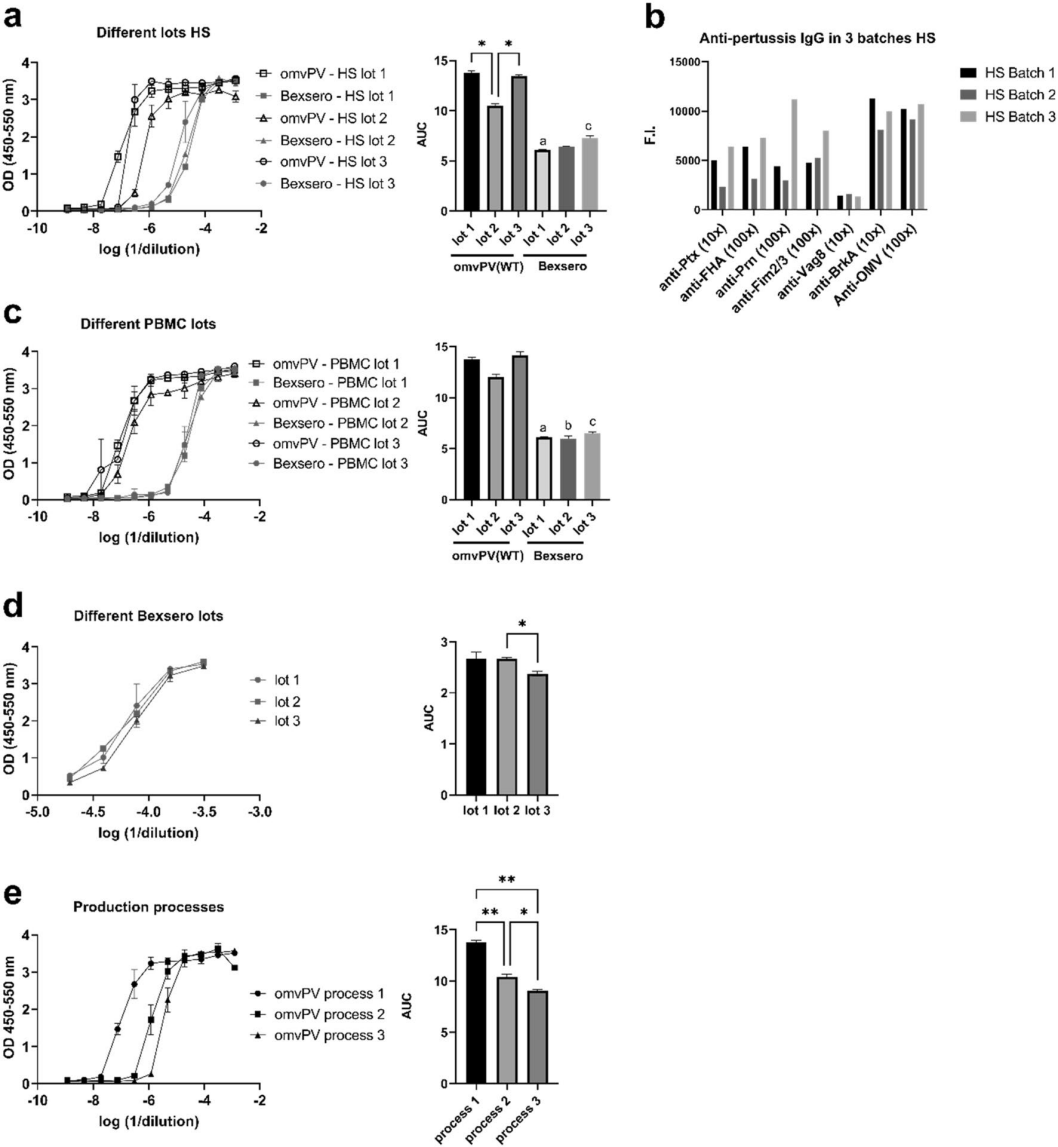
Robustness of HS-based MAT using omvPV(WT LPS) and Bexsero. The robustness of the HS-based MAT for omvPV(WT LPS) was assessed with different batches of HS, PBMCs and production processes. (**a**) The IL-6 responses were compared using 3 different HS lots combined with PBMC lot #A and is expressed as optical density (OD) and as area under the curve (AUC) of the OD curves. Each sample was tested in duplo. (**b**) Levels of immunoglobulin G (IgG) antibodies directed against Ptx, Prn, FHA, Fim2/3, BrkA, Vag8 and OMV were determined in 3 different HS lots. Results are expressed as fluorescence intensities (F.I.). (**c**) The IL-6 responses were compared using 3 different PBMC lots combined with HS lot #1. (**d**) The IL-6 responses of 3 different Bexsero lots were compared. (**e**) The IL-6 responses of 3 different omvPV(WT LPS) production processes were compared. The area under the curve (AUC) of the fitted curves was determined and depicted as mean ± standard deviation (SD). *p < 0.05, **p < 0.01, a—significantly lower than lot 1 omvPV(WT LPS), b—significantly lower than lot 2 omvPV(WT LPS), c—significantly lower than lot 3 omvPV(WT LPS).

To determine the robustness of the HS-based MAT, two additional HS lots were tested. These batches showed comparable IL-6 responses to the responses obtained with the first HS batch (lot 1; same as in Fig. 1) (Fig. 2A). HS lot 2 gave significantly lower IL-6 responses than lot 1 or 3 for omvPV(WT LPS) (*p* value is 0.018). The use of HS in the MAT could better reflect the actual situation following immunization in humans. However, depending on the antigen, previous immunizations and/or natural infections that occurred in the donor(s) could lead to the presence of antibodies in the human serum source that may affect the MAT results. Especially for *B. pertussis* it is known that most individuals have a certain level and diversity of anti-pertussis antibodies^24^, while presence of anti-NmB antibodies is less likely (except after Bexsero immunization). To that end, we decided to determine the presence of different anti-pertussis antibodies in the three batches of HS using a panel of antigens that was available (Fig. 2B). Firstly, these results indicated that all three batches of HS indeed contained anti-pertussis antibodies against all antigens tested. Secondly, it was observed that the antibody levels differed among the three batches. This indicates that HS will contain anti-pertussis antibodies and that each batch varies.

Subsequently, the robustness of the MAT was investigated by comparing three different lots of cryopreserved pooled PBMCs, each lot contains PBMCs from 4 different donors. IL-6 response curves of the two additional PBMC lots were similar to those of lot A (lot A; same as in Fig. 1) (Fig. 2C). There were no significant differences between PBMC lot A, B and C within a sample group (Bexsero or omvPV(WT LPS)), only significant differences between sample groups. Finally, the variety of three lots of the reference vaccine Bexsero was determined Here, lot 2 showed comparable reactogenicity to lot 1 while lot 3 had a lower reactogenicity (Fig. 2D).

At this stage, no robustness could be performed on three identical pertussis OMV lots as the optimization of the production process was still ongoing. Therefore, as alternative, the MAT assay was used to test omvPV products obtained with three different production processes that were aiming at a higher OMV purity and yield. These results revealed that the production process can affect the reactogenicity as the omvPV from process 1 was the most reactogenic while process 3 resulted in the lowest reactogenicity (Fig. 2E) indicating that the MAT can also be used in this stage of vaccine development.

### MAT applicable to test and compare pyrogen-containing vaccines including OMVs from different bacterial species

**Figure 3:**
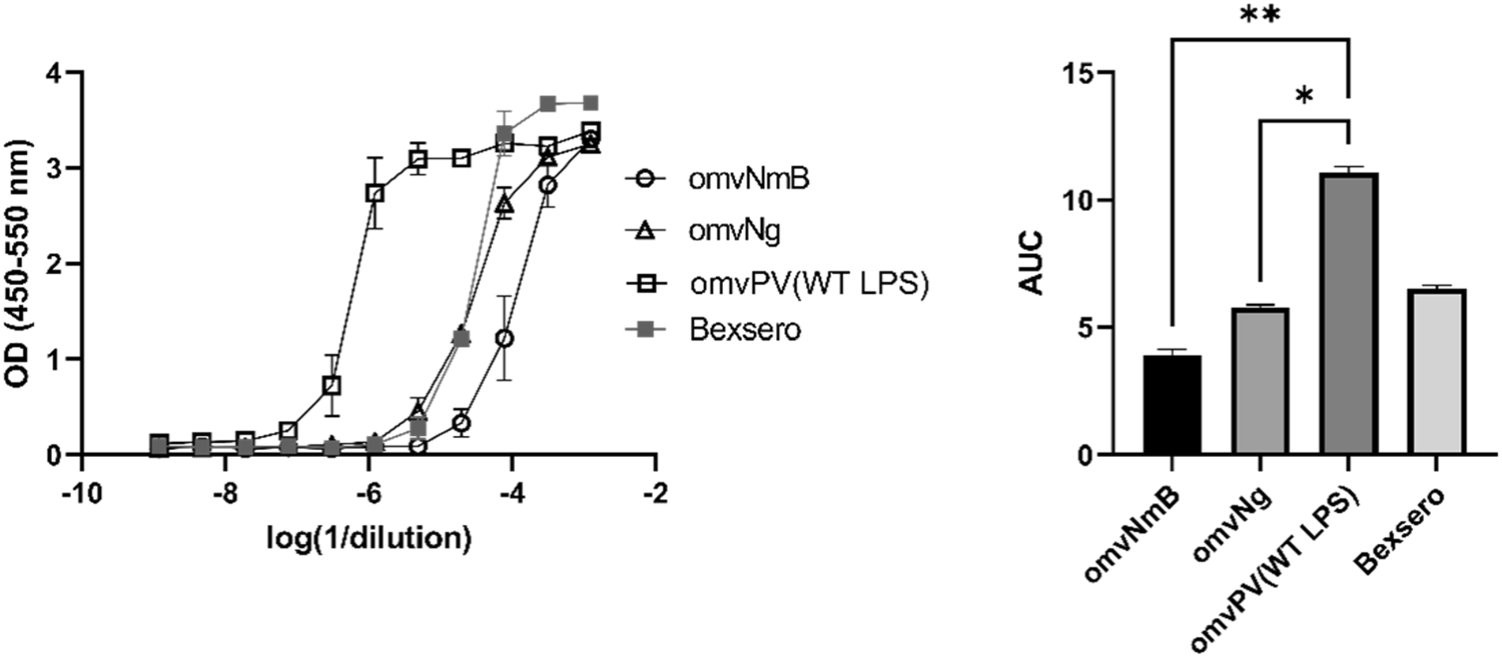
Reactogenicity towards OMVs from different bacterial species. The IL-6 responses were compared for different product dilutions of omvNmB(Lpxl1), omvNg(Lpxl1) and omvPV(WT LPS), which were all set at 100 μg/mL protein and are expressed as OD (left graph) and AUC of the OD curves (right graph). In addition, Bexsero was used at the human dose and this contained 50 μg/mL of OMVs (in 0.5 mL). The AUC is depicted as mean ± standard deviation. *p < 0.05, **p < 0.01.

The developed MAT was applied to test and compare the reactogenicity of OMV vaccines targeting different bacterial species which are in various stages of development (Fig. 3). Next to the omvPV(WT LPS), used for the MAT optimization, OMV vaccines against *Neisseria meningitis* subclass B (omvNmB(Lpxl1)) and *Neisseria gonorrhoeae* (omvNg(Lpxl1)) were included. Both were isolated from strains with a modified LPS structure to reduce reactogenicity. For all OMV vaccines, 100 µg/mL of protein was used as estimated starting dose. This experiment demonstrated that of all these products the omvPV(WT LPS) had indeed the highest reactogenicity profile in the MAT. Both omvNmB(Lpxl1) and omvNg(Lpxl1) at 100 µg/mL revealed a similar or even lower reactogenicity profile as compared to Bexsero. Remarkably, the shape of the IL-6 response curves was similar for OMVs from different bacterial species, while the composition and structures of PAMPs may differ between species. Overall, these results indicated that the optimized MAT was applicable for testing multiple OMV-containing vaccines.

### IL-6 responses by PBMCs caused by combination of LPS and NEPs present in OMVs

After the initial MAT optimization studies, omvPV became available with a modified LPS omvPV(LpxA). Hence, a direct comparison could be made between the omvPV(WT LPS) and omvPV(LpxA). In addition, we decided to isolate LPS from both strains to solely determine the effect of the LPS modification in the MAT. By dosing the OMVs on LPS content instead of protein concentration a comparison could be made with the purified LPS to assess the contribution of LPS to the reactogenicity in the OMVs and whether other PAMPs may play a role.

**Figure 4:**
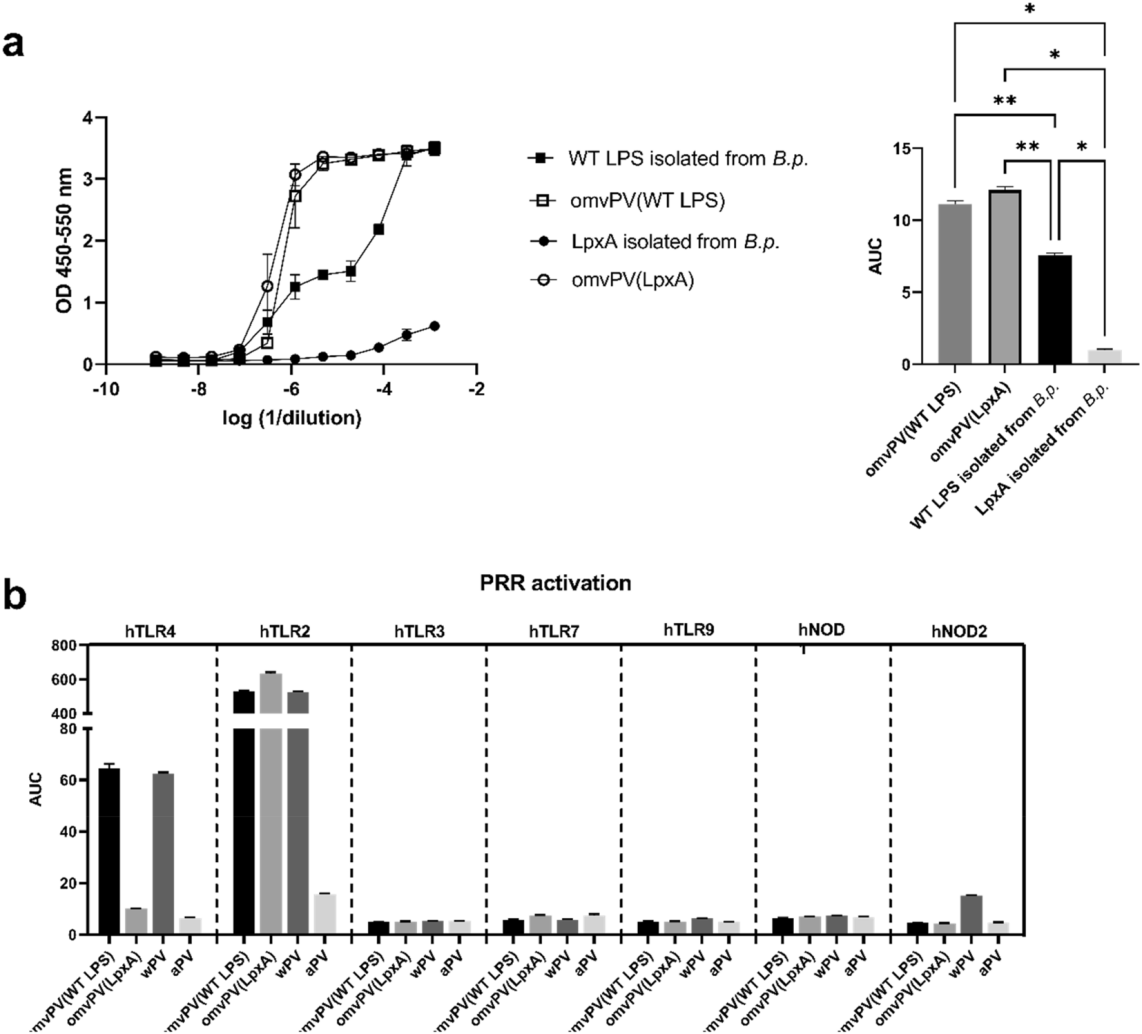
Contribution of LPS and other PAMPs in reactogenicity of pertussis OMVs. (**a**) The IL-6 responses by PBMCs were compared after stimulation with OMVs and isolated LPS of omvPV(WT LPS) and omvPV(LpxA) strains. 100 μg/mL WT LPS and detoxified LPS (LpxA) were isolated from representative *B. pertussis* strains, and this was compared to omvPV(WT LPS) and omvPV(LpxA) which had a concentration of 100 μg/mL LPS instead of 100 μg/mL protein. (**b**) The activation of different PRRs by pertussis OMVs was compared to other pertussis vaccines. Different reporter cell lines were stimulated with omvPV(WT LPS), omvPV(LpxA), wPV or aPV in duplo using a dilution range of the vaccines (2 – 0.000128 µg/mL). Area under the curve was calculated and depicted as mean ± standard deviation.

The LpxA modification in the LPS clearly reduced the reactogenicity of the purified LPS sample as the LpxA isolate induced less IL-6 production compared to the WT LPS isolate (Fig. 4A). However, the shape of the IL-6 response curve of the WT LPS isolate suggests that there are still other PAMPs present in the LPS preparation, since this curve was not a standard S-shape as expected for LPS. This could be due to non-pyrogen free materials used during the LPS isolation procedure or that some non-endotoxin pyrogens (NEPs) from *B. pertussis* were purified together with LPS.

Strikingly, the effect of the LPS modification was not observed when comparing both the omvPV(WT LPS) and omvPV(LpxA) as both products provided equal reactogenicity profiles (Fig. 4A). Importantly, the IL-6 response to both LPS isolates was lower than to the two omvPV preparations at equal LPS concentrations, which indicated that the reactogenicity caused by both omvPV is not solely caused by the presence of LPS. Therefore, these data indicate that the omvPV preparations contain other NEPs from the bacteria next to LPS that contribute to reactogenicity in the HS-based MAT.

To determine which other PAMPs were present in the omvPVs we tested the activation of single pathogen recognition receptors (PRRs) using human reporter cell lines using wPV and aPV as control (Fig. 4B and Supplementary Figure 1). First, these experiments confirmed that the LpxA modification in the omvPV(LpxA) resulted in an ablated TLR4 activation comparable to the level induced by aPV. TRL4 activation was observed at a similar level after stimulation with omvPV(WT LPS) or wPV. TLR2 activation was remarkably high after stimulation with both OMV vaccines and the wPV. Moreover, the TLR2 activation by omvPV(LpxA) was higher as compared to omvPV(WT LPS) and wPV. As expected, no TLR2 or 4 activation was observed after aPV stimulation. The other PPR's tested (TLR3, TLR7, TLR9, NOD1 and NOD2) were not activated by any of the vaccines except for some minor NOD2 activation by wPV. Overall, we can conclude that the pertussis OMVs and wPV cause TLR2 activation because of the lipoprotein content next to TRL4 activation by LPS. TLR2 activation by omvPV seems even the only remaining PRR activation, of the tested PRRs, when the TLR4 activation is ablated.

### Comparison between MAT and RPT

Finally, the two omvPV variants were compared in different doses to different reference vaccines (Bexsero and wPV) using both the MAT and the RPT to bridge differences in species (human vs. rabbit) as well as in vivo and in vitro results. For the MAT and RPT the same samples were used. Start concentrations of 100 µg/mL or 20 µg/mL total protein for omvPV(WT LPS) and omvPV(LpxA) were used in the MAT, whereas 0.5 mL of these solutions were injected intramuscular (i.m.) in the RPT resulting in a dose of 50 µg or 10 µg per rabbit, respectively.

**Figure 5:**
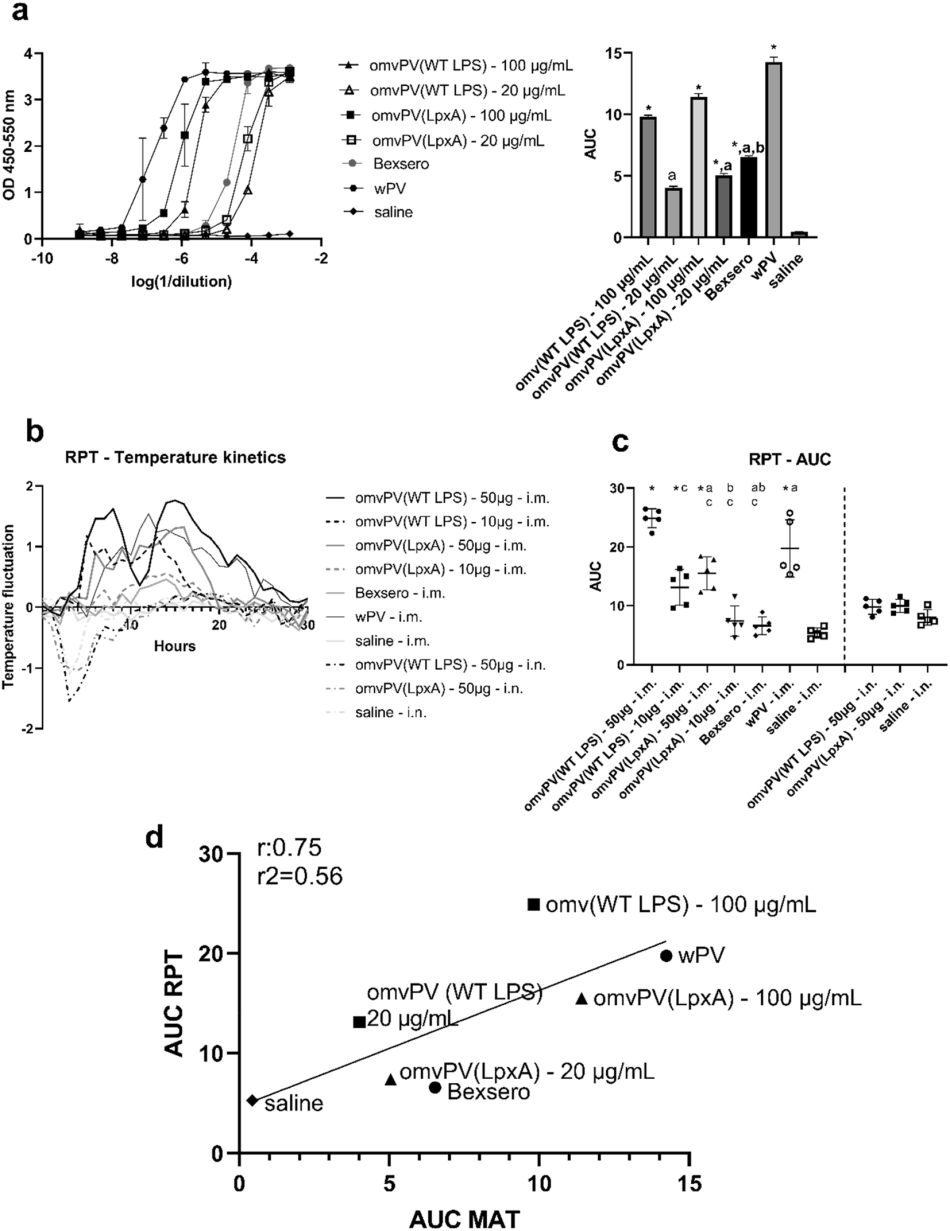
Comparison of pyrogenicity of pertussis OMVs after LPS modification or change in dosing in MAT and RPT. (**a**) Comparison of two different doses (dose 1 = 50 µg, dose 2 = 10 µg) of the omvPV(LpxA) and omvPV(WT LPS) to placebo (NaCl), wPV (WHO standard), and Bexsero in MAT. Results representing IL-6 concentration are either depicted as OD 450–550 nm in a dilution range or as AUC per group with SD. (**b**) Temperature fluctuations in rabbits measured every hour over a period of 30 h after immunization as compared to temperature before immunization. (**c**) Area Under the Curve (AUC) from t = 0 (immunization) to 30 h after immunization was calculated. *Significantly higher than the saline control; a—significantly lower compared to 50 µg omvPV(WT LPS); b—significantly lower compared to 10 µg omvPV(WT LPS); c—significantly lower compared to wPV. For each group, the mean and SD are depicted as well as data from each individual rabbit (N = 5 per group). (**d**) Correlation between MAT and RPT. The AUC results from the MAT were plotted against the AUC results from the RPT (i.m. administration only). The different groups are presented as  omvPV(WT LPS),  omvPV(LpxA),  Bexsero and wPV,  saline including a regression line with R = 0,75 and R^2^ = 0.56.

First, in vitro pyrogenicity was determined in the MAT. Here, the induction of IL-6 of two different concentrations (100 or 20 µg/mL) of the omvPV(LpxA) and omvPV(WT LPS) were compared to that induced by placebo (NaCl), wPV (WHO standard), and Bexsero (Fig. 5A). The results showed that all vaccines induced more IL-6 as compared to saline. The wPV induced the highest level of IL-6. The 100 µg/mL concentrations of both omvPV(LpxA) and omvPV(WT LPS) were less pyrogenic as compared to the wPV but more pyrogenic as compared to Bexsero. The 20 µg/mL concentrations of both omvPV(LpxA) and omvPV(WT LPS) were less pyrogenic as compared to Bexsero. Remarkably, although also observed in the earlier experiment, the omvPV(WT LPS) seemed less pyrogenic as compared to the omvPV(LpxA) for both doses.

Secondly, for the rabbit pyrogenicity study a similar experimental setup was chosen as performed in the MAT. Rabbits received 50 or 10 µg of omvPV(WT LPS) or omvPV(LpxA) i.m. and body temperature was compared to that of placebo (NaCl), wPV, and Bexsero treated rabbits. The body temperature after immunization was corrected for the basal body temperature measured during the 5 days before immunization. Subsequently, the fluctuations in temperature were monitored over a period of 30 h (Fig. 5B), the maximum increase in body temperature after immunization was determined (Supplementary Figure 2), and the AUC of the corrected body temperature after immunization was calculated for each rabbit (Fig. 5C).

Importantly, i.m. administration of the omvPV(LpxA) with both the 50 µg and 10 µg dose resulted in a significantly lower maximum body temperature increase and smaller AUC compared to the omvPV(WT LPS) (Fig. 5C and Supplementary Figure 2), which indicates that the LPS modification leads to a safer reactogenicity profile for the omvPV in rabbits. A dose–response effect was observed when 50 µg omvPV(LpxA) was compared to 10 µg omvPV(LpxA). The low dose induced a significantly smaller increase in maximum body temperature (*p* = 0.003) and AUC (*p* < 0.001). Moreover, the AUC was lower after i.m. immunization with 50 µg or 10 µg omvPV(LpxA) when compared to wPV immunization. However, the maximum increase in body temperature did not differ between the 50 µg dose and wPV. Moreover, both maximum temperature increase, and AUC were higher for 50 µg omvPV(LpxA) when compared to i.m. saline administration. Strikingly, the 10 µg omvPV(LpxA) dose did not differ from the saline group in terms of maximum temperature increase and AUC.

For omvPV(WT LPS), both the high and low dose induced a higher maximum body temperature increase and a greater AUC when compared to the saline group (Fig. 5B,C and Supplementary Figure 2). When the effect of the 50 µg dose and 10 µg of the omvPV(WT LPS) were compared, the low dose resulted in a significantly smaller increase in maximum body temperature (*p* = 0.02) and AUC (*p* < 0.001). Comparison of 50 µg i.m. omvPV(WT LPS) with wPV showed that both vaccines induced a similar maximum body temperature increase, but the AUC was higher after omvPV(WT LPS) immunization. Also, a comparable maximum body temperature increase was found after administration of 10 µg omvPV(WT LPS), however in this dose the AUC of the omvPV(WT LPS) was lower compared to wPV. Finally, Bexsero induced a slight increase in maximum body temperature, but the AUC did not differ significantly from the saline control. Summarized, both doses of the omvPV(WT LPS) and wPV as well as the 50 µg dose of omvPV(LpxA) induced a higher body temperature increase (both in maximum increase and in AUC) compared to Bexsero when administered i.m. in rabbits.

Next to intramuscular immunization, we also evaluated intranasal (i.n.) administration for both omvPV as we previously revealed that this route of immunization provided even more promising immunogenic and protective profiles as compared to systemic immunization with pertussis OMVs^26^. Remarkably, after intranasal immunization, in contrast to i.m. immunization, no rise in temperature was observed nor any differences between groups (Fig. 5B,C and Supplementary Figure 2). Notably, for approximately the first 10 h the temperature was decreased, which could be a result of the anaesthesia that was only applied for i.n. administration (Fig. 5B). Overall, these results demonstrate that it is possible to obtain a desired safety profile in rabbits with the omvPV. For intramuscular immunization, the LPS modification and lower dosing are required while the intranasal route may allow higher dosing and/or does not necessitate a LPS modification.

Finally, the correlation between the AUC results of the MAT and RPT (i.m. administration) was determined as the same products were tested in both tests. The correlation analysis showed a positive correlation (r = 0.75, *p* value = 0.05) between AUC of the MAT and the AUC of the RPT (Fig. 5D). In addition, when the decrease in temperature and MAT reactogenicity between 100 and 20 µg/mL (corresponding to 50 and 10 µg/rabbit) were compared for omvPV(WT LPS) and omvPV(LpxA) the decreases seems to be parallel to each other, confirming a good correlation between MAT and RPT results for different omvPVs. Overall, this shows that the in vitro and in vivo test results were comparable and that the MAT can serve as an in vitro alternative for assessing the reactogenicity of OMV based vaccines.

## Discussion

This study showed that the MAT is robust and can be used to determine reactogenicity in different OMV-based preparations, even those which are still in the developmental phase. The MAT method used in this study is a reference lot comparison method, which is the preferred method for inherent pyrogenic products according to Ph. Eur. chapter 2.6.30. However, a reference lot is usually not available for vaccines in early development and therefore it was decided to use Bexsero as a reference lot. Bexsero is a multicomponent OMV-containing vaccine with a proven safety profile and sufficient literature on MAT and Bexsero is available^13–15^. The results for Bexsero found in this study were in line with the data reported by Valentini et al. even though there were differences in the MAT format used; the IL-6 curves obtained with our cryopreserved pooled PBMCs started to decrease from dilution 3200x (− 3.5 log) and this is comparable to the IL-6 results obtained by Valentini et al. with cryopreserved individual donors^13^. This gave confidence that the MAT was fit for purpose. The MAT can be performed with several serum sources, such as FBS or HS. Both sources were tested, and no significant differences were observed between FBS or HS for omvPV(WT LPS) or Bexsero, however the HS-based assay seemed to be somewhat more responsive after stimulation with OMVs. This is probably because OMV consists of multiple components from Gram-negative bacteria. Previous results from our laboratory have shown that the FBS-based MAT is more sensitive towards endotoxin detection while HS-based MAT is more sensitive towards non endotoxin pyrogens^27^. Replacing FBS with HS also results in an animal component free assay. Together with the fact that HS-based MAT better reflects the human immune system it was decided to use HS-based MAT for further testing of the MAT with omvPV(WT LPS).

The comparison between omvPV(WT LPS) dosed at 100 μg/mL and the human dose of Bexsero (containing 50 μg/mL omvNmB, three recombinant protein antigens and aluminium hydroxide) in HS-based MAT showed that this initial dose of omvPV(WT LPS) obtained from mice studies was much more reactogenic than Bexsero. This difference may be due to different expression of PAMPs in the OMV products since they are derived from different bacteria. We showed that the reactogenicity of our omvNmB was comparable to Bexsero. Moreover, the presence of the Lpxl1 modification present in Bexsero and omvNmB could cause a reduced reactogenicity^28^. In addition, the presence of aluminium hydroxide in Bexsero, which is not present in omvPV(WT LPS), omvPV(LpxA), omvNg or omvNmB, may also affect the level of reactogenicity. Lowering the dose of omvPV(WT LPS) to 20 μg/mL resulted in IL-6 responses which were comparable to Bexsero.

We have shown that during (early) development of an OMV-based vaccine the MAT can be performed with a reference lot (in this case Bexsero) which is different from the product under test. However, at later stages each OMV type should get their own reference batch instead of Bexsero. Because often a non-related reference lot yields IL-6 curves which are not perfectly parallel to the tested lot; or the maximum IL-6 release obtained with the tested and the reference lot are different. This may create difficulties when comparing the tested samples with the reference lot through a parallel line assay for e.g., routine consistency testing of the products. When a pyrogenic content needs to be determined for batch release, it is even more important to move to a reference lot which is similar to the product since it is possible that upon changing of HS lot or PBMC lot the reactogenicity of the tested samples, but not that of the non-related reference lot is influenced or vice versa and this could impact the pyrogenic content assessment.

This study revealed that for most products the reactogenicity profile obtained with the RPT could be reflected with the MAT showing that this assay could serve as an alternative for animal testing. However, strikingly, this study also demonstrated that the MAT may reflect the human in vivo situation more closely and therefor reveal reactogenicity profiles that would not have been detected by the RPT. In this study, the expected reduction in reactogenicity of the LpxA modification in the omvPV compared to the omvPV(WT LPS) was not observed in the MAT while this reduction was observed in the RPT. Purified LpxA was however less reactogenic as compared to purified WT LPS which proves that the LPS modification could be detected in the MAT. This reduced reactogenicity of purified LpxA in comparison to WT LPS was previously shown in the RPT^17^. That study also showed that the reactogenicity of purified LpxA was at the same desired level as that of aPV. The higher activation of TLR2 by the omvPV(LpxA), likely due to more or different lipoproteins composition on the outer membrane, may cause a higher IL-6 reaction by the human PBMCs toward omvPV(LpxA) and potentially explain why no difference was observed in the MAT despite the presence of the LPS modification. Moreover, humans may be more sensitive for TLR2 agonists compared to rabbits, as shown by Schindler et al. for IL-1 in a whole blood MAT setting^29^. The TLR2 and TLR4 activation shown in this study by omvPV(WT LPS and omvPV(LpxA) was previously also observed for OMV containing vaccines from NmB after i.m. administration in mice^28^.

A new pertussis vaccine candidate based on outer membrane vesicles was used in this study to optimize the MAT and compare to the RPT. This improved pertussis vaccine is designed to induce a broader immunity profile in terms of antigen-targeting, antibody response and T cell response^3,30^ as compared to the current aPV^19,31^. However, the vaccine should have a comparable safety profile to the aPV and consequently be much less reactogenic as current wPV. The current study proved for the first time, with both the RPT and the MAT, that the omvPV has indeed a desired safety profile in terms of systemic reactogenicity. This was achieved by either modifying the LPS, changing the dosing, administrating intranasally or a combination of these. Importantly, especially with the omvPV(LpxA) but also with omvPV(WT LPS) different strategies are possible that result in a less reactogenic profile as compared to wPV. This finding is in line with what we previously observed in our in vivo mouse model where the omvPV(WT LPS) induced less profound pro-inflammatory responses as compared to the more reactogenic whole-cell vaccine whilst maintaining the same type and level of immunity and protection^3^. It should be noted that the international wPV standard that was used in this study is not adjuvated with alum as most commercial combination vaccines containing wPV are. The suggested dosing of 100 µg/mL total protein for intramuscular immunization with the omvPV(WT LPS) is however too reactogenic in comparison to i.e., Bexsero, yet, lowering the dose five times leads to a favourable safety profile. Further research should confirm whether at this dose immunogenicity and protective capacity remain intact. Interestingly, no fever was observed in the RPT for both types of OMV when a high dose was administered intranasally, the results were comparable to the saline group. A higher tolerability for reactogenicity at mucosal sites might explain this observation. This may indicate that for mucosal administration a broader dosing range can be tested as systemic reactogenicity seems of lesser concern. Mucosal administration with pertussis OMVs also leads to better protection compared to systemic immunization as previously demonstrated by our group^25,26^. Based on the comparison from this study we can also conclude that for mucosal vaccine administration the MAT unfortunately cannot replace the RPT as it cannot mimic the local administration. And although systemic reactogenicity seems absent in the RPT after intranasal immunization with omvPV, local safety concerns should be further investigated using other in vivo or in vitro tests. Combined, the current findings favour the promising approach of mucosal immunization with pertussis OMVs, however, also systemic immunization with OMVs at lower doses or with an LPS modification can be applied as an improved pertussis vaccine.

Overall, we can conclude from this study that the adjusted MAT can be applied in multiple stages, of the vaccine development chain e.g., screening of experimental vaccines, monitoring vaccine reactogenicity prior to clinical trials (pre-clinical stages) and for batch releases during the clinical stage, to determine the reactogenicity of pyrogen-holding vaccines such as the OMV vaccines. The MAT can easily and swiftly be applied to supply first indications of reactogenicity in preliminary stages of vaccine development when the RPT is too labour-intensive and costly. Moreover, modifications to the vaccine to reduce reactogenicity can be screened rapidly with this assay. For final products, the MAT can serve as a valuable assay next to, or eventually as replacement of, the RPT to assess the reactogenicity of pyrogen-holding vaccines. Finally, with the MAT, combined with results from the RPT, we proved that our pertussis vaccine based on outer membrane vesicles has a safe reactogenicity profile, which is required for an improved pertussis vaccine.

### Supplementary Information

Supplementary Information.

## Data Availability

The data that support the findings of this study are available from the corresponding author upon reasonable request.
