# Supplementary material for: In vitro alternative for reactogenicity assessment of outer membrane vesicle based vaccines
Source: Sci Rep. 2023 Aug 4;13:12675. doi: 10.1038/s41598-023-39908-7 (PMC10403550; doi:10.1038/s41598-023-39908-7)
Supplement: Supplementary file 1 — Supplementary Information. [file 41598_2023_39908_MOESM1_ESM.docx]

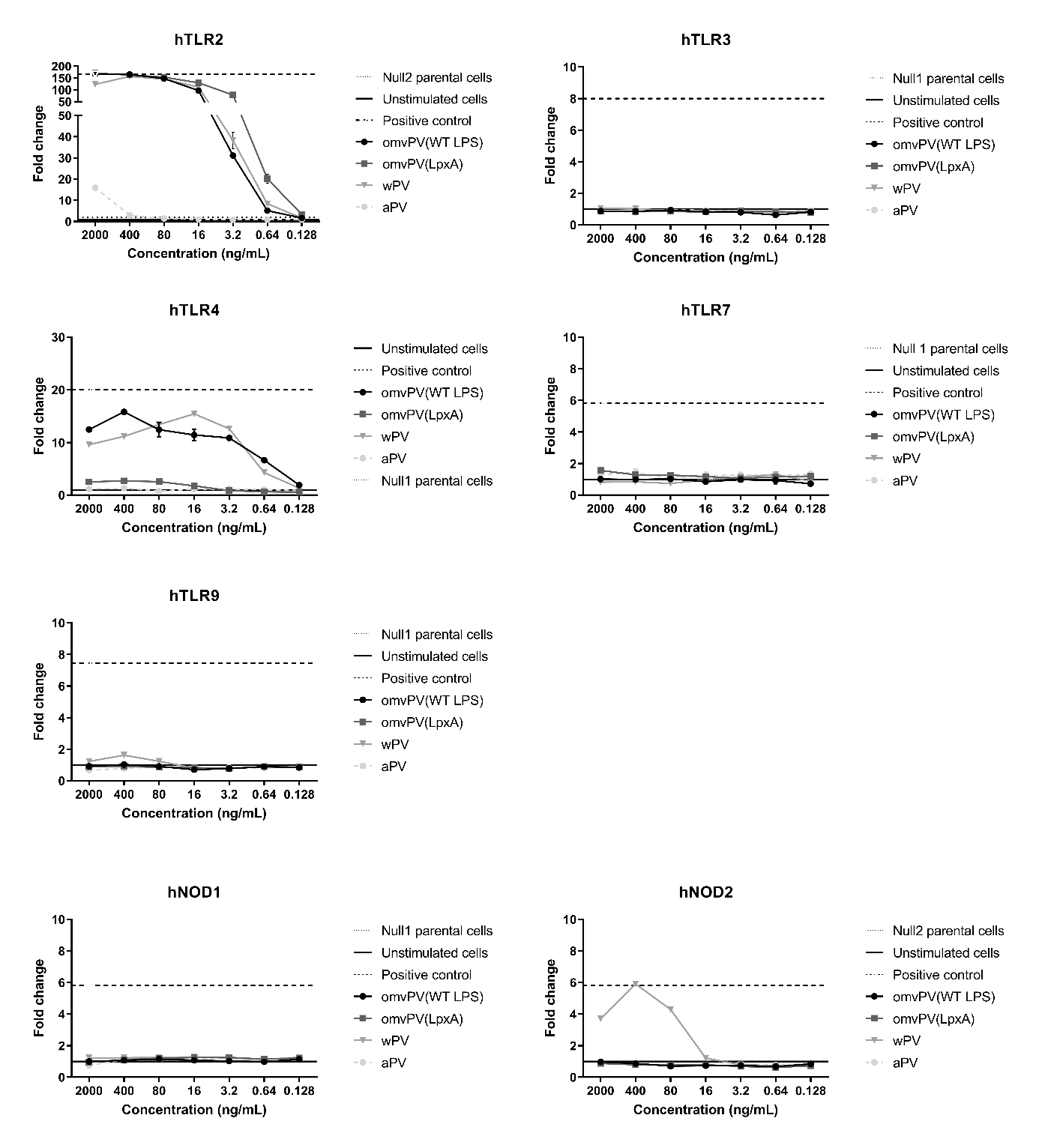


**Supplementary Figure 1 - PRR activation by different pertussis vaccines.** Different reporter cell lines were stimulated with omvPV(WT LPS), omvPV(LpxA), wPV or aPV in duplo using a dilution range of the vaccines (2 – 0,000128 µg/mL). Single data points from stimulation with a positive control, unstimulated and the Null1 or Null2 parental cells are depicted as one line. Fold change is calculated in comparison to stimulation of control cell line.


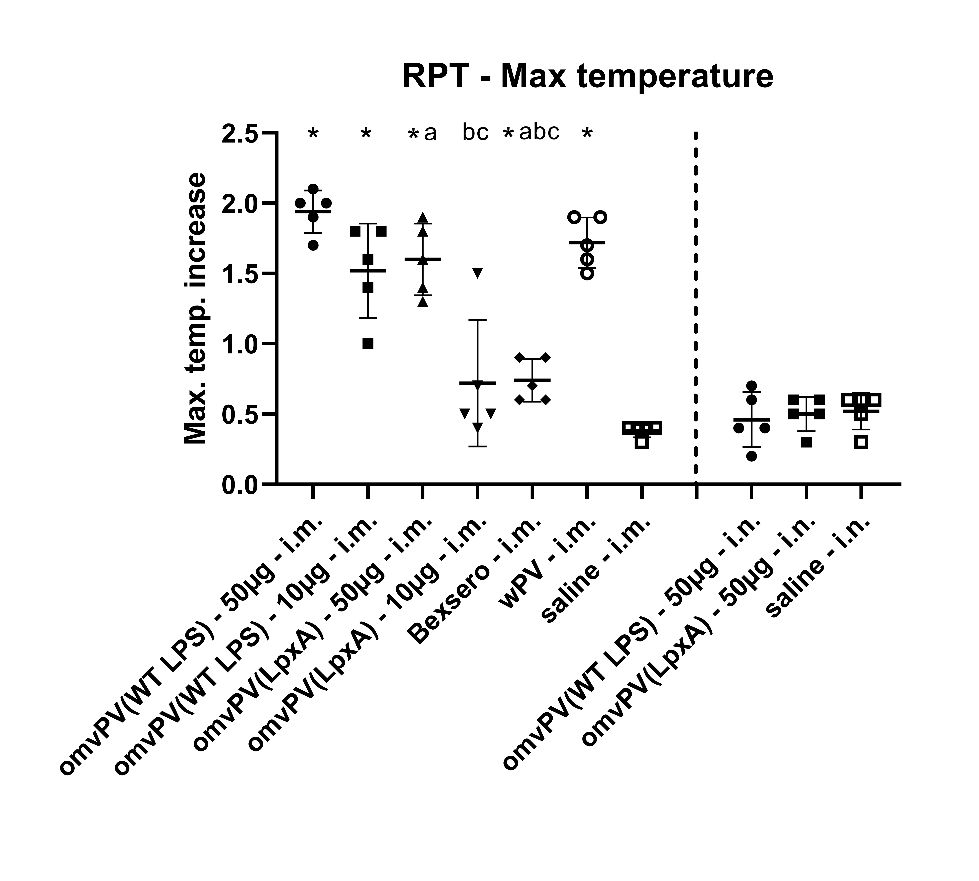


**Supplementary Figure 2 -**  Maximum increase in body temperature of the rabbits after immunization was determined over a 30 hours period.
